# SIRT1 deficiency interferes with membrane resealing after cell membrane injury

**DOI:** 10.1371/journal.pone.0218329

**Published:** 2019-06-26

**Authors:** Daisuke Fujiwara, Naotoshi Iwahara, Rio Sebori, Ryusuke Hosoda, Shun Shimohama, Atsushi Kuno, Yoshiyuki Horio

**Affiliations:** 1 Department of Pharmacology, Sapporo Medical University School of Medicine, Sapporo, Japan; 2 Department of Neurology, Sapporo Medical University School of Medicine, Sapporo, Japan; University of Minnesota Medical School, UNITED STATES

## Abstract

Activation of SIRT1, an NAD^+^-dependent protein deacetylase, ameliorates muscular pathophysiology of δ-sarcoglycan-deficient TO-2 hamsters and dystrophin-deficient *mdx* mice. We found that SIRT1 was highly expressed beneath the cellular membranes of muscle cells. To elucidate functional roles of SIRT1 on muscles, skeletal muscle-specific SIRT1 knockout mice (SIRT1-MKO) were generated. SIRT1-MKO mice showed muscular pathology similar to mild muscular dystrophies with increased numbers of centrally nucleated small myofibers and decreased numbers of middle-sized (2000–3001 μm^2^) myofibers compared to those of wild-type (WT) mice. Accordingly, SIRT1-MKO mice showed significantly decreased exercise capacity in treadmill and inverted hanging tests with higher levels of serum creatine kinase activities compared with those in WT mice. Evans blue dye uptake after exercise was greater in the muscles of SIRT1-MKO than those of WT mice, suggesting membrane fragility in SIRT1-MKO mice. Because SIRT1 was dominantly localized beneath the membranes of muscular cells, SIRT1 may have a new role in the membranes. We found that levels of fluorescent FM1-43 dye intake after laser-induced membrane disruption in C2C12 cells were significantly increased by SIRT1 inhibitors or *Sirt1-siRNA* compared with those of control cells. Inhibition of SIRT1 or SIRT1-knockdown severely disturbed the dynamic aggregation of membrane vesicles under the injured site but did not affect expression levels of membrane repair proteins. These data suggested that SIRT1 had a critical role in the resealing of membrane-ruptured muscle cells, which could affect phenotypes of SIRT1-MKO mice. To our knowledge, this report is the first to demonstrate that SIRT1 affected plasma-membrane repair mechanisms.

## Introduction

Cycles of contraction and relaxation in skeletal muscles and cardiac cells induce cellular membrane friction and strain that could cause membrane rupture. Plasma membrane disruption is rapidly resealed by membrane repair mechanisms for cell survival [1]. Membrane resealing is triggered by Ca^2+^ influx through the injured site, where Ca^2+^ activates Ca^2+^ binding proteins including calpain 3, which is involved in the resealing of membrane structures through their Ca^2+^-dependent protease activity [1]. Accordingly, mutations in the calpain 3 gene cause limb girdle muscular dystrophy type 2A [2]. F-actin is accumulated promptly at the site of membrane disruption and annexins, phospholipid-interacting proteins with Ca^2+^-binding activity, are also recruited to the injured site and contribute to membrane repair [3]. Dysferlin interacts with negatively charged phospholipids in a Ca^2+^-binding manner and its genetic defects result in limb girdle muscular dystrophy type 2B [4]. After membrane injury, intracellular small vesicles containing dysferlin are recruited to the injured site and form a large vesicle to reseal membranes [1]. Dysferlin interacts with mitsugumin 53 (MG53) and caveolin 3, which are also essential to repair membrane damage [5]. Mutations of caveolin 3 cause limb girdle muscular dystrophy 1C [6] and MG53 knockout mice show dystrophic phenotype [7].

An NAD^+^-dependent protein deacetylase SIRT1, one of seven members of the sirtuin family, is a mammalian homologue of yeast Sir2 (silent information regulator 2), the overexpression of which elongates yeast lifespan [8]. SIRT1 localizes in nuclei and regulates gene expression through deacetylation of nuclear proteins such as histones and transcriptional factors [8]. Because SIRT1 is a nucleo-cytoplasmic shuttling protein [9], it also regulates cytosolic proteins including autophagic components. SIRT1 is expressed in the skeletal muscle cells where it increases insulin sensitivity [10], improves mitochondrial function [11], promotes a fiber shift from fast- to slow-twitch muscles and decreases expression levels of atrophy genes [12].

Overexpression of SIRT1 in the skeletal muscles of *mdx* mice, a mouse model of *Duchenne* muscular dystrophy, ameliorates muscular pathology, decreases blood creatine kinase (CK) levels and increases exercise performance [12]. We have shown that pharmacological activation of SIRT1 by oral administration of resveratrol ameliorates pathological phenotypes of skeletal muscles and hearts, decreases serum CK levels and improves muscular and cardiac functions in *mdx* mice [13–17]. SIRT1 activated by resveratrol decreases cellular oxidative stress levels by increasing SOD2 expression levels [18], suppressing NADPH oxidase expression [13], and promoting autophagy of damaged mitochondria via activation of FOXOs [16,17]. Resveratrol inhibits the development of tissue fibrosis via promoting deacetylation and degradation of transcriptional co-activator p300 [14], and increases expression levels of muscle myosin heavy chains and troponins [13], which may be regulated by deacetylation and activation of transcriptional co-activator PGC1α [8]. However, it remains unclear whether membrane repair in the muscle cells is regulated by SIRT1.

In the present study, we found that a substantial amount of SIRT1 was expressed beneath the cellular membranes of skeletal muscle cells, suggesting that SIRT1 might have an additional role in the plasma membranes. To elucidate the function of SIRT1, we generated skeletal muscle-specific SIRT1 knockout (SIRT1-MKO) mice and found that they were prone to suffer from exercise-induced muscle injury and had a mild dystrophic phenotype. We show here that SIRT1 is indispensable in the membrane resealing after injury in C2C12 cells.

## Materials and methods

### Animal models

All animal experiments were conducted according to The Animal Guideline of Sapporo Medical University and approved by the Animal Use Committee of Sapporo Medical University. Animals were housed in the conventional condition under adequate temperature (24 ± 2°C) and humidity (50 ± 5%) under a 12 h light /12 h dark cycle with access to food and water *ad libitum*.

Skeletal muscle-specific SIRT1 knockout mice were generated by crossing floxed SIRT1 mice [19] (SIRT1^flox/flox^, strain name B6;129-Sirt1tm1Ygu/J) with human α-skeletal muscle actin promoter driven Cre mice (ACTA1-Cre79Jme/J), both obtained from the Jackson Laboratory. Genotypes were confirmed by PCR using primers for Cre (forward: 5’-CGAATAACTACCTGTTTTGCCGGGT-3’, reverse: 5’-TCGCCATCTTCCAGCAGGCGCACCA-3’) and for SIRT1 flox alleles [19].

### Histopathology and immunohistochemistry

Mice were deeply anesthetized by intraperitoneal injection of xylazine (10 mg/kg) and pentobarbital sodium (50 mg/kg), killed by decapitation and then their muscles were isolated by well-trained persons. Transverse cryosections (10 μm thick) prepared from the quadriceps muscle were stained with hematoxylin and eosin (HE) or Masson’s trichrome (MT) using standard procedures. For immunohistochemistry, cryosections were fixed in cold methanol/acetone (50/50, v/v) for 15 min, treated with Triton X-100 and then blocked with 3% bovine serum albumin in phosphate buffered saline (PBS). Sections were incubated with antibodies against SIRT1 (1:1000 dilution; ab110304, Abcam, Cambridge, UK or 07–131, Merck Millipore, Massachusetts, USA), anti-dystrophin (1:1000 dilution; ab15277, Abcam), anti-caveolin 3 (1:1000 dilution; ab30750, Abcam), anti-nNOS (1:500 dilution; A-11, Santa Cruz, Texas, USA) and anti-CD31 (1:100 dilution; ab56299, Abcam) overnight at 4°C. Sections were then probed with secondary antibodies of an anti-mouse IgG antibody conjugated with Alexa Fluor 488 or 594 (1:2000 dilution; Thermo Fisher Science, Waltham, USA), an anti-rabbit IgG antibody conjugated with Alexa Fluor 594 (1:2000 dilution; Thermo Fisher Science) or an anti-rat IgG antibody conjugated with Alexa Fluor 647 (1:2000 dilution; Thermo Fisher Science). Sections were also stained with Hoechst33342 (Dojindo, Kumamoto, Japan), phalloidin-FITC (Sigma Aldrich, St Louis, USA) or wheat germ agglutinin conjugated with Alexa Fluor 594 (WGA, Thermo Fisher Science).

### Immunoblot analysis

For Western blot analysis, C2C12 cells and muscle tissues were homogenized with Mammalian Cell Lysis Buffer (Sigma Aldrich) and Cell Lysis Regent for mammalian tissue (Sigma Aldrich), respectively. For subcellular fractionation, skeletal muscle tissue (quadriceps) was homogenized by a polytron homogenizer in 40 volumes (w/v) of buffer A (50mM Tris-HCl, ph 7.5, 1 mM DTT, 1 mM EDTA) containing protease inhibitor cocktail (Nacalai tesque, Tokyo, Japan). The homogenates were then centrifuged for 30 min at 100,000 x g to obtain the membrane pellet and cytosol fractions. The pellet fraction was resuspended and homogenized in 40 volumes of buffer A containing 1% NP40 (Sigma Aldrich).

Western blot analysis was performed as described previously [13,14]. The following antibodies were used: anti-SIRT1 (1:1000 dilution; ab110304, Abcam), anti-dystrophin (1:1000 dilution; ab15277, Abcam), anti-caveolin 1 (1:1000 dilution; ab2910, Abcam), anti-caveolin 3 (1:1000 dilution; ab30750, Abcam), anti-nNOS (1:200 dilution; A-11, Santa Cruz), anti-Histone H3 (1:2000 dilution; ab1791, Abcam), anti-Histone H3 acetyl K9 (1:10,000 dilution; ab4441, Abcam), anti-GAPDH (1:2000 dilution; MAB374, Sigma Aldrich) and anti-α-tubulin (1:2000 dilution; T5168, Sigma Aldrich).

### RNA analysis

The total RNA was extracted using an RNeasy Fibrous Tissue Mini Kit (Qiagen, Valencia, USA) for skeletal muscle tissues and an RNeasy Mini Kit (Qiagen) for other cell lysates. Reverse transcriptase reactions were performed with the Go Script Reverse Transcription system (Promega, Madison, USA). For quantitative PCR, cDNA was analyzed by StepOne Real-Time PCR systems (Applied Biosystems, Foster City, USA) using GoTaq qPCR Master Mix (Promega). Each sample was run in duplicate, and the mean value was used to calculate the mRNA levels of the gene of interest. All data were normalized to 18S ribosomal RNA using the standard curve method. The following primer sequences were used: 5’-GACGCTGTGGCAGATTGTTA-3’ and 5’-GGAATCCCACAGGAGACAGA-3’ for mouse SIRT1, 5’- CGGACAGGATTGACAGATTG -3’and 5’- CAAATCGCTCCACCAACTAA -3’ for mouse 18S ribosomal RNA, 5’- TGGGAACTACGGGAACAAG-3’ and 5’- AGTGGCATCCATCAAAGACC-3’ for mouse dysferlin, 5’- CCTCCTTTTCAAGGTTGCAG-3’ and 5’- TGGATGCTGGGATTATAGCC-3’ for mouse MG53, 5’- AGTCAATGATGCAGGCTTCC-3’ and 5’- CATGTGTTTGTCCGCATAGC-3’ for mouse calpain 3, 5’- ATTCTGGGCTCCTGAAAGTG-3’ and 5’-TCGAGCGGTCCTTAATATCG-3’ for mouse cofilin-2, 5’- AAGGTGTGGATGAAGCAACC-3’ and 5’- AAGGGCTTTCCATTCTCCTG-3’ for mouse annexin A1 (ANNA1), 5’- ACATTGCCTTCGCCTATCAG-3’ and 5’- AAAATCACCGTCTCCAGGTG-3’ for mouse annexin A2 (ANNA2), 5’- AAGCCTGTTGAAAGGACTGG-3’ and 5’- TGATCTTGGCGAGACTGTTG-3’ for mouse acid sphingomyelinase (ASM), 5’- ACTTGACAAAGGAGGACCTGAG-3’ and 5’-ATTTTGTCCACAGCCAGAGG-3’ for mouse S100A10, 5’- TTGTACCGTGCATCAAGAGC-3’ and 5’- AAAGAGTGGATCGCAGAAGG-3’ for mouse caveolin 1, 5’- TCAACGATACCAGCCACAAG-3’ and 5’- TCTCCTTGCAGTGAATGTCC-3’ for mouse caveolin 3, and 5’-ATCTTGTCGGGCTTTCCAC-3’ and 5’-ATCCAAAGGCTTTCCCAGAT-3’ for utrophin.

### Treadmill test

Exercise capacity of WT and SIRT1-MKO mice at 3 months of age (n = 12 for both) and 30 months of age (n = 6 for both) was measured using a motor-driven treadmill system (MK-680S, Muromachi Kikai, Tokyo, Japan). The slope of the treadmill was kept constant at 5°, and the speed was increased stepwise as follows: 5 min at 10 m/min, 1 min at 11 m/min, 1 min at 12 m/min, 1 min at 13 m/min, 1 min at 14 m/min, 30 min at 15 m/min, 1 min at 16 m/min, 1 min at 17 m/min, 1 min at 18 m/min, 1 min at 19 m/min and finally 20 m/min until exhaustion. Exhaustion was defined as spending >50% of the time in a stage or >3 consecutive seconds on the shock grid. All experiments were performed by investigators blinded to mouse genotypes.

### Inverted hanging test

Inverted hanging tests were performed after the treadmill exercise, because mice could hang for an extremely long time without exercise. The slope of the treadmill was kept constant at 0°, and the speed was increased stepwise as follows: 5 min at 10 m/min, 2 min at 12 m/min, 2 min at 14 m/min, 10 min at 16 m/min, 10 min at 18 m/min and finally 20 m/min until exhaustion. After the treadmill exercise, a mouse was placed on a net that was then inverted. The hanging time of the mouse was analyzed with 1 min elapsing between each of five determinations per mouse and the average of the hanging times was calculated. WT and SIRT1-MKO mice at 5 months of age (n = 6 for both) were analyzed. All experiments were performed by investigators blinded to mouse genotypes.

### Forelimb grip strength test

Fore arm grip strength was assessed by using a grip strength meter (GPM-100B, MELQUEST, Toyama, Japan). Experiments were performed by the same operator, who was blinded to the genotypes of the mice. Grip strength of WT and SIRT1-MKO mice at 5 months of age (n = 5 for both) was analyzed with 1 min elapsing between each of three determinations per mouse. All experiments were performed by investigators blinded to mouse genotypes.

### Myofiber damage evaluation

WT and SIRT1-MKO mice at 5 months of age were injected intraperitoneally with 1% Evans blue dye (EB, FUJIFILM Wako Pure Chemicals) in PBS 16 h before the treadmill exercise and were then subjected to treadmill running as described above (Treadmill test) to induce muscle injury. The quadricepses were sampled from mice 1 h after treadmill running and were rapidly frozen. WT (n = 4) and SIRT1-MKO (n = 4) mice were analyzed. Frozen muscles were embedded in an optimal cutting temperature compound (Tissue-Tek, Torrance, USA), and were cross-sectioned at 5 μm by cryostat. To visualize the plasma membranes, sections were stained with WGA conjugated with Alexa Fluor 488 (Thermo Fisher Scientific) in accordance with the manufacture’s protocol. WGA-Alexa Fluor 488 (green) and EB (red) were observed by a confocal laser microscopy (LSM510META, ZEISS, Oberkochen, Germany). The EB-positive cell areas divided by total cross-section area were compared between WT and SIRT1-MKO mice. Percentage of EB positive fibers of SIRT1-MKO mice was also compared with that of WT mice. ImageJ was used to analyze the area positive for EB.

### Assessments of CK and LDH activities

Mice at 5 months of age were subjected to treadmill running as described above (Treadmill test) to induce muscle injury by exercise. To measure serum activities of CK and lactate dehydrogenase (LDH) as markers of muscle injury, blood samples of mice were obtained by snipping their tails 14 days before and 1 h after treadmill exercise. Serum CK and LDH activities were assessed with CYGNUS AUTO CK (Shino-Test Corporation, Tokyo, Japan) and Quickauto-neo LD (Shino-Test Corporation), respectively, in LABSPECT 008 HITACHI Automatic Analyzer (Hitachi, Tokyo, Japan). WT (n = 11) and SIRT1-MKO mice (n = 8) were analyzed.

### Cell culture

C2C12 cells were cultured in Dulbecco’s modified Eagle’s medium with high glucose (Nacalai Tesque, Kyoto, Japan) containing 10% fetal bovine serum (MP Biomedicals, Aurora, USA) in an incubator with 5% CO_2_ set at 37°C. Twenty-four h after passage, cells were treated with Vehicle, 10 mM nicotinamide (NAM; FUJIFILM Wako Pure Chemicals) or Ex527 (Tocris Bioscience, Ellisville, USA) and incubated for 12 h. RNAi-mediated knockdown was performed by transfection of *Sirt1-siRNA* (Sigma-Aldrich, Mm_Sirt1_5675) or *Control-siRNA* (Sigma-Aldrich, Mission_SIC-001) (30 nM for both) targeting SIRT1 using Lipofectamine RNAiMAX Transfection Reagent (Thermo Fisher Scientific) according to the manufacturer’s instruction. Experiments were completed at 48 h after transfection. For the differentiation, C2C12 cells were cultured in Dulbecco’s modified Eagle’s medium with low glucose (FUJIFILM Wako Pure Chemicals) containing 2% house serum (Thermo Fisher Scientific) for 1 week.

### Membrane repair assay

Just before the assay, the medium was changed to Live Cell Imaging Solution (Thermo Fisher Scientific) containing 10 μM FM1-43 dye (Thermo Fisher Scientific) at 37°C. Cell membrane damage was induced with a Nikon A1 laser scanning confocal microscope equipped with plan Apo 100x oil immersion objective lens (NA 1.4). For laser injury, a 1 μm x 1 μm area was irradiated by a 405 nm laser at 100% power by using the photo-activation mode. Each irradiation time was 395.92 msec and irradiation was repeated 5 times (total 20 sec). Images were captured by a 488 nm laser. Images were acquired for 20 sec every 5 sec before injury, just after every injury or after all injuries, and for 4 min every 5 sec following the injury. For each image, fluorescence intensity of a cell was measured by Nikon NIS Elements v4.1 software. To analyze fluorescence intensity of a differentiated myotube, the portion of the myotube irradiated with a laser in a microscopic field was analyzed. Data is presented as change of fluorescence intensity relative to the value at 0 sec (ΔF/F0). Data from at least six myoblast cells or four myotubes were compared in one experiment, and confirmed with three independent experiments.

### Statistical analyses

Data are expressed as means ± SEM. Statistical significance was determined using an unpaired Student’s two-tailed t-test for two data sets. Two-way repeated measures ANOVA and the Student-Newman-Keuls post hoc test were used to analyze differences in data between WT and SIRT1-MKO mice before and after the treadmill exercise. For all tests, p<0.05 was considered statistically significant. When comparing data from the two groups, the sample size required for statistical power to be 0.8 was estimated in the settings with an alpha value at 0.05 and standard deviation of data from the control group in each experiment. All analyses were performed with SigmaStat (Systat, San Jose, USA).

## Results

### Skeletal muscle-specific SIRT1 knockout (SIRT1-MKO) mice showed a mild dystrophic phenotype

**Fig 1:**
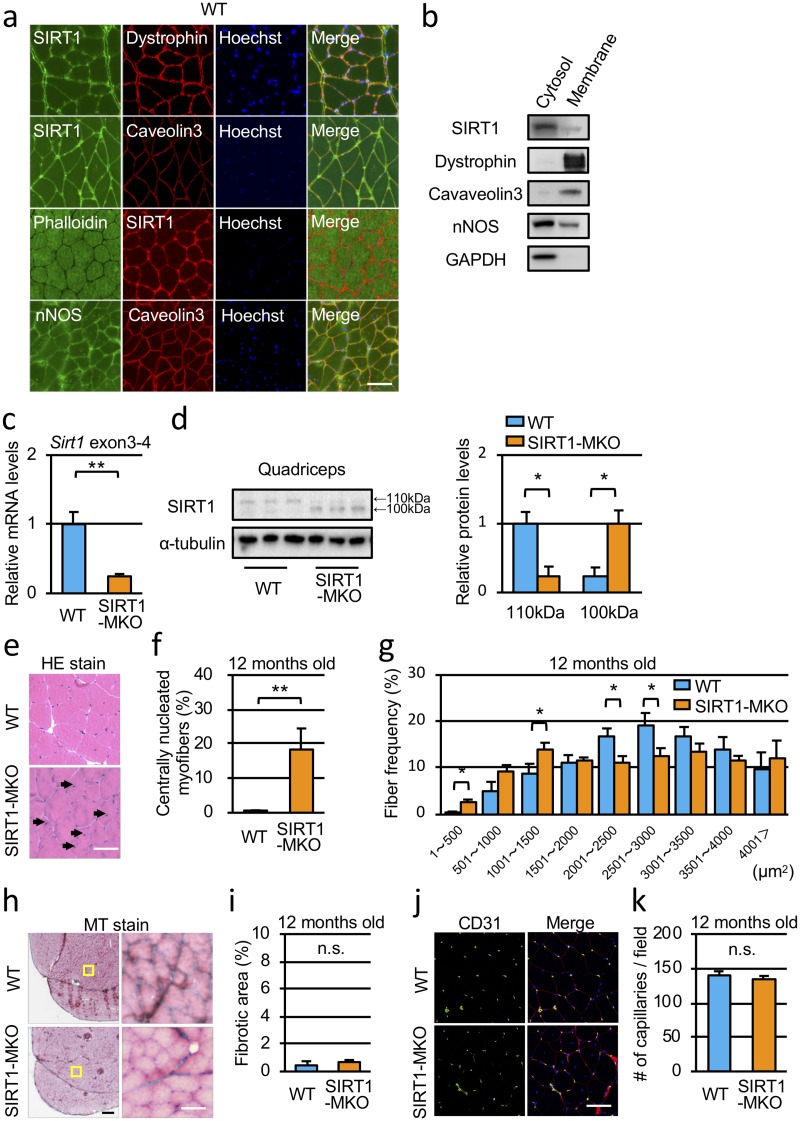
SIRT1-MKO mice have mild dystrophic pathology. (a) Immunofluorescence staining for SIRT1 (ab110304, Abcam), dystrophin, caveolin 3 and nNOS in muscle sections from WT mice at 6 months of age. Cytoplasm of skeletal muscles was stained with phalloidin. (b) Immunoblots of cytoplasmic fraction and membrane fraction of quadricepses from WT mice. (c) Levels of *Sirt1* mRNA containing exon 3 and exon 4 in quadriceps of WT and SIRT1-MKO mice at 12 months of age were analyzed by qPCR using primers amplifying the region (n = 3). (d) Immunoblots of quadriceps from WT and SIRT1-MKO. The band of WT SIRT1 is detected around 110 kDa, whereas the band of mutant SIRT1, which defects amino acid sequence derived from *Sirt1* gene exon 4, is found at 100 kDa. Immunoblot for α-tubulin was used as a loading control. (e) Hematoxylin and eosin (HE) staining of quadriceps from WT (top) and SIRT1-MKO (bottom) mice at 12 months of age. The arrows indicate centrally nucleated myofibers. (f) Frequency of centrally nucleated myofibers in quadriceps from WT and SIRT1-MKO mice (n = 3 for both). (g) Cross-sectional area of myofibers in the quadriceps muscles of WT and SIRT1-MKO mice (n = 3 for both). (h) Masson’s trichrome (MT) staining of quadriceps from WT (top) and SIRT1-MKO (bottom) mice at 12 months of age. (i) The ratio of fibrotic areas of WT and SIRT1-MKO mice (n = 3 for both). (j) ImmunofIuorescence staining for CD31 (green), visualizing blood vessels, performed using quadriceps from WT (top) and SIRT1-MKO (bottom) mice at 12 months of age. WGA lectin staining (red) was used for visualization of the connective tissues in skeletal muscles. (k) The number of capillary blood vessels stained with an anti-CD31 antibody was equivalent between WT and SIRT1-MKO mice. Scale bars of images are 50 μm (a, e, h right and j) and 200 μm (h left), respectively. Data are represented as means ± SEM. Significant difference was determined by a two-tailed Student’s *t*-test: *p<0.05, **p<0.001. n.s., not significant.

To identify the intracellular distribution of SIRT1 in the muscle, we stained sections of mouse quadriceps with a SIRT1 antibody (Fig 1a). Costaining of SIRT1 with membrane (dystrophin and caveolin 3) or cytoplasmic (actin staining by phalloidin) markers, and nuclei (Hoechst 33342) showed that high levels of SIRT1 expression were detected in the nuclei, cytosol and also beneath cellular membranes (Fig 1a). Immunostaining using another antibody against SIRT1 also showed that SIRT1 was found beneath plasma membranes (Part D in S1 Fig). To confirm the localization of SIRT1, Western blot analysis of subcellular fractions of quadricepses was performed. As shown in Fig 1b, both of the cytoplasmic and membrane fractions contained SIRT1, although the intensity of SIRT1 band in the membrane fraction was less than that in the cytoplasmic fraction.

To identify the function of SIRT1 beneath sarcolemma, we generated skeletal muscle-specific exon 4-deleted SIRT1 knockout mice by crossing SIRT1 flox mutant mice (SIRT1^flox/flox^) with transgenic mice carrying a Cre transgene under the control of a human α-skeletal muscle actin promoter. In the quadricepses of SIRT1-MKO mice, expression levels of SIRT1 mRNA containing exon 4 were reduced to 30% of those of WT mice (Fig 1c). In SIRT1-MKO mice, mutant SIRT1 of about 100 kDa, which lacked amino acid sequence coded by exon 4 of the *Sirt1* gene, was expressed (Fig 1d). Localization of the mutant SIRT1 in quadricepses from SIRT1-MKO mice was quite similar to that of wild type SIRT1 in immunostaining (Part A in S1 Fig), indicating that the deleted amino acid sequence of SIRT1 did not affect subcellular localization of SIRT1. HE staining of quadricepses showed that SIRT1-MKO mice had myofibers with central nuclei (Fig 1e: arrows). Nearly 20% of myofibers in the quadriceps of SIRT1-MKO mice had central nuclei, whereas only 0.5% of myofibers in WT mice had central nuclei (Fig 1f). Fiber frequencies of small fibers less than 500 μm^2^ increased in quadricepses of SIRT1-MKO mice compared with those of WT mice and the number of middle-sized fibers (2001–3000 μm^2^) in SIRT1-MKO mice decreased compared with those in WT mice (Fig 1g). Accordingly, fiber splitting and fiber regeneration were frequently detected in the muscles of SIRT1-MKO mice (Parts B and C in S1 Fig). MT staining of quadricepses showed no difference in fibrosis between WT and SIRT1-MKO mice (Fig 1h and 1i). In addition, necrotic fibers were rarely observed in the muscles of SIRT1-MKO mice. Because SIRT1 has been reported to control blood vessels growth [20], we assessed the number of capillaries. However, the number of CD31-positive capillaries in the section of quadricepses was similar between WT and SIRT1-MKO mice (Fig 1j and 1k).

### Muscle fragility with reduced exercise endurance and strength in SIRT1-MKO mice

**Fig 2:**
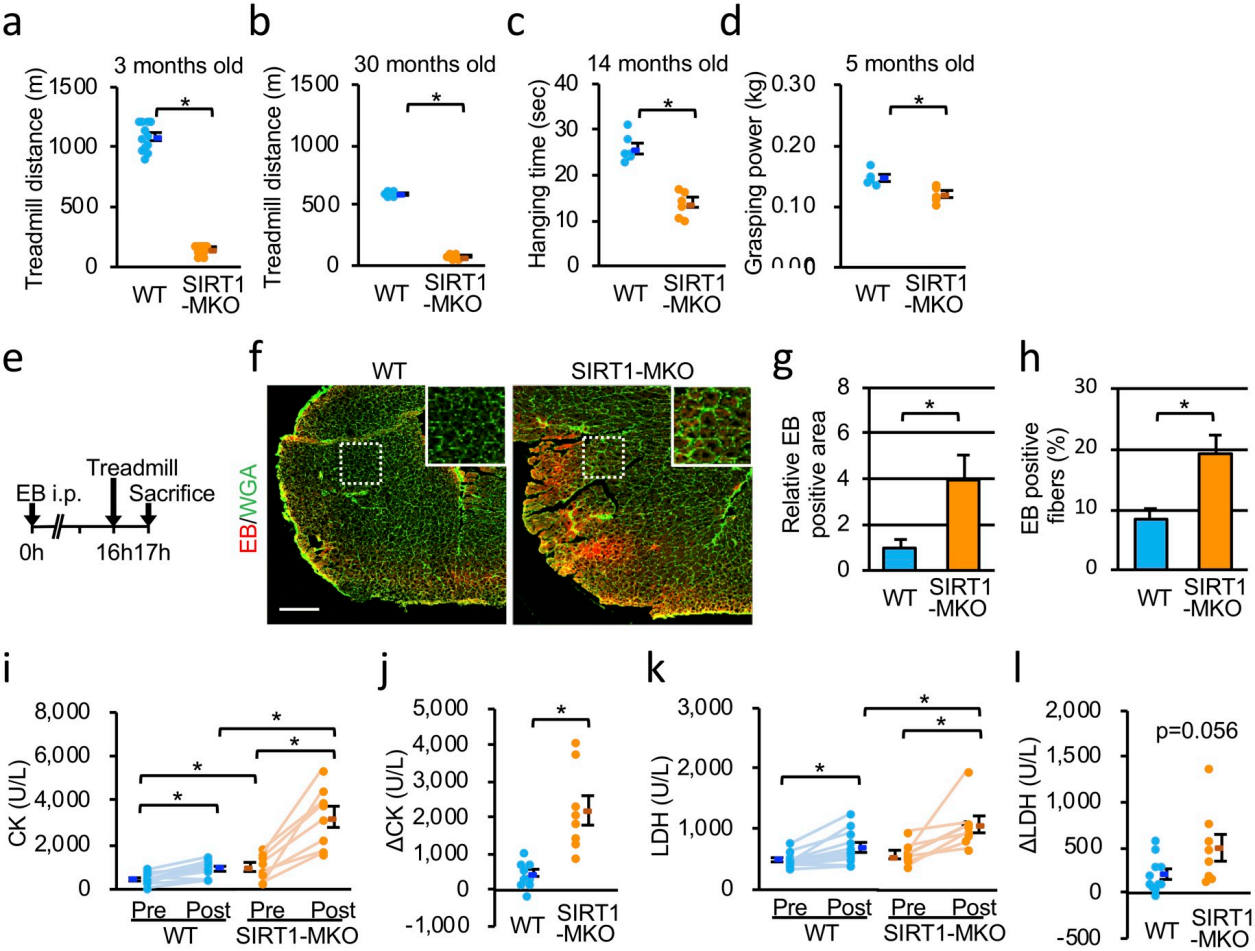
SIRT1-MKO mice have muscle fragility with reduced exercise endurance and strength. (a, b) Treadmill activity of WT and SIRT1-MKO mice at 3 months of age (a: n = 12) and 30 months of age (b: n = 6). (c) Hanging time after treadmill exercise of WT and SIRT1-MKO mice at 12 months of age (n = 6). (d) Grasping power of WT and SIRT1-MKO mice at 5 months of age (n = 5). (e) A schema of Evans blue dye (EB) uptake experiments. (f) Representative images of EB (red) uptake into quadriceps myofibers of WT (left) and SIRT1-MKO (right) mice 1 h after treadmills exercise. Scale bar is 500 μm. (g) The EB-positive area of WT and SIRT1-MKO mice (n = 4). (h) The percentage of EB-positive fibers of WT and SIRT1-MKO mice (n = 4). (i) Serum creatine kinase activities (CK) of WT and SIRT1-MKO mice before (Pre) and after (Post) treadmill experiments. (k) Serum lactate dehydrogenase activity (LDH) of WT and SIRT1-MKO mice before (Pre) and after (Post) treadmill experiments. (j and l) The differences in CK (j: ΔCK) and LDH (l: ΔLDH) activities before and after treadmill experiments (n = 11 and 8 in WT and SIRT1-MKO mice, respectively). Data are represented as means ± SEM and significant differences in (a)-(d), (g), (h), (j) and (l) were determined by a two-tailed Student’s *t*-test. *p<0.05. Two-way repeated measures ANOVA and Student-Newman-Keuls post hoc tests were performed to determine significant differences in (i) and (k).

Previously, exercise activities were reported to decrease significantly in skeletal muscle-specific SIRT1 deficient mice [12]. In the present study, exercise performance of male 3 and 30 months old male WT and SIRT1-MKO mice was examined. The treadmill running showed that SIRT1-MKO mice could run for a much shorter distance than WT mice (Fig 2a and 2b). Duration of hanging after the treadmill exercise in 14-month-old SIRT1-MKO mice was significantly shorter than that of WT mice of the same age (Fig 2c). Grasping power was weaker in SIRT1-MKO mice than WT mice and levels of grasping power of SIRT1-MKO mice were approximately 80% of those of WT mice at the age of 5 months (Fig 2d). Because body weights (BW) were comparable in WT and SIRT1-MKO mice (Parts A-D in S2 Fig), these data suggested that SIRT1 was indispensable for maintaining exercise endurance and muscle strength. To examine whether SIRT1-MKO mice were prone to muscle injury when compared with WT mice, SIRT1-MKO mice and WT mice were injected intraperitoneally with EB before a 16 h of treadmill test and examined 1 h after the exercise (Fig 2e). EB enters into and is retained in a cell only when the cell membranes have a rupture. In our study, EB fluorescence levels and the percentage of EB-positive fibers in quadricepses of SIRT1-MKO mice were much higher than those of WT mice, indicating that quadricepses of SIRT1-MKO mice had much more exercise-induced muscle damage compared than those of WT mice (Fig 2f–2h). We found that levels of serum CK and LDH activities in SIRT1-MKO mice were higher than those of WT mice before exercise and much more CK and LDH were liberated from muscles of SIRT1-MKO mice after the treadmill exercise (Fig 2i–2l). Liberating amounts of CK through exercise were much higher in SIRT1-MKO mice compared with those of WT mice, indicating that muscles of SIRT1-MKO mice were prone to being damaged by exercise (Fig 2j).

### SIRT1 is necessary for membrane repair in C2C12 cells

**Fig 3:**
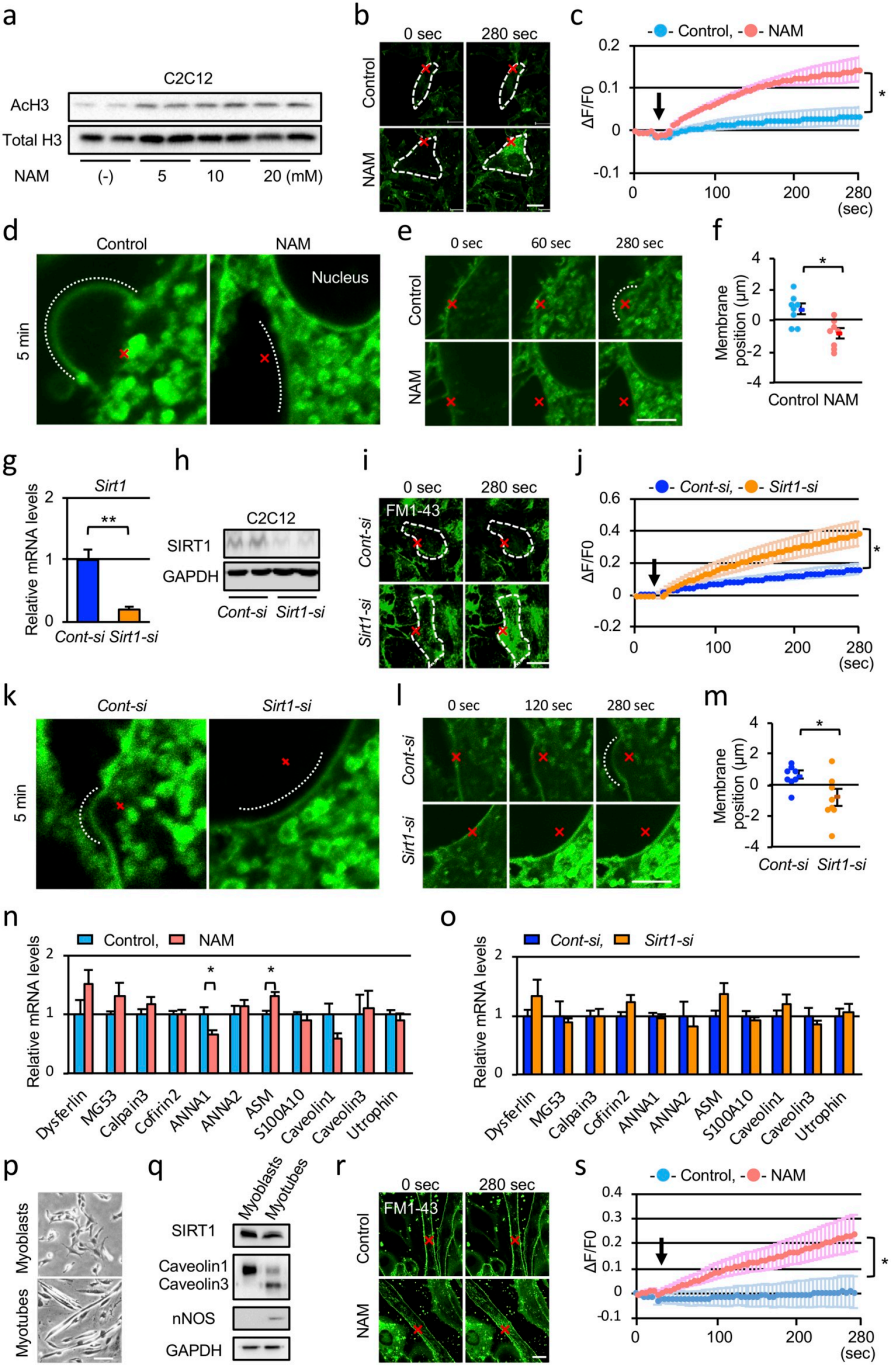
SIRT1 inhibition and SIRT1 knockdown inhibit membrane resealing in C2C12 cells. (a) Immunoblots of acetylated (top) and total (bottom) histone H3 in C2C12 cells 12 h after treatment with various concentrations of nicotinamide (NAM). (b) Plasma membrane repair kinetics upon laser injury measured by membrane impermeable FM1-43 dye influx (green). Representative images before and after laser injury of control (top) and NAM (bottom) treated C2C12 cells. X-marks (red) indicate laser injury points and dotted lines (white) indicate cellular shapes. (c) Time course of FM1-43 dye influx after laser injury in C2C12 cells treated with PBS or NAM (n = 6). Arrow indicates the time point of membrane injury. (d) Enlarged images of the cells 5 min after laser injury. The dotted curves indicate convex (Control) and concave (NAM) membrane 5 min after laser irradiation, respectively. (e) Representative high-power field images of C2C12 cells before and after laser injury. (f) Movement of the membrane at the injured site after irradiation. Membrane positions 4 min after laser injury in C2C12 cells treated with PBS or NAM (n = 8). (g, h) Knockdown of *Sirt1 mRNA* levels (g, n = 3) and decrease of SIRT1 protein levels (h) by *Sirt1-siRNA* (*Sirt1-si*). (i) Representative images before and after laser injury of C2C12 cells treated with *Cont-si* and *Sirt1-si*. (j) Time course of FM1-43 dye influx over time after laser injury in C2C12 cells treated with *Cont-si* or *Sirt1*-*si* (n = 8). Arrow indicates the time point of membrane injury. (k) Enlarged images of the cells 5 mins after laser injury. The dotted curves indicate convex (*Cont-si*) and concave (*Sirt1-si*) membrane 5 min after laser irradiation. (l) Representative high-power field images of C2C12 cells treated with *Cont-si* and *Sirt1-si*. (m) Membrane positions 4 min after laser injury in C2C12 cells treated with *Cont-si* and *Sirt1-si*. (n = 8). (n, o) mRNA levels of membrane repair proteins in C2C12 cells treated with NAM (n) or *Sirt1-si* (o) were compared with those of control cells (n = 3). (p) Morphology of C2C12 myoblasts and myotubes. (q) Immunoblots of C2C12 myoblast cells and differentiated myotubes. (r) Representative images before and after laser injury of control (top) and NAM-treated myotubes (bottom). (s) Time course of FM1-43 dye influx after laser injury in control and NAM-treated myotubes (n = 4). Scale bars are 20 μm (b and i), 5 μm (d, e, k and l) and 100 μm (r), respectively. Data are represented with means ± SEM. Significant differences were determined by a two-tailed Student’s *t*-test. *p<0.05, **p<0.001.

Muscular fragility of SIRT1-MKO mice suggested that SIRT1 might have a protective role in muscle cells beneath sarcolemma (Fig 1a). Muscular membrane fragility is caused by genetic mutations of the dystroglycan complex and also of membrane resealing machinery such as dysferlin [21, 22]. There have been no previous reports focusing on how SIRT1 interacts with the dystroglycan complex or membrane repair proteins. Because phenotypes of SIRT1-MKO mice were similar to those of dysferlinopathy (see Discussion), we examined whether membrane resealing in C2C12 myoblast cells was affected by SIRT1 inhibition. Membrane resealing can be monitored by an influx of fluorescent dye FM1-43 in cells after laser irradiation. NAM is an inhibitor of SIRT1 and 5 mM or more doses of NAM inhibited deacetylation of histone H3 (H3K9) in C2C12 cells (Fig 3a). When cells were treated with 10 mM NAM, FM1-43 uptake by cells was significantly promoted (Fig 3b lower panels, S2 Video) compared with those of control cells (Fig 3b upper panels, S1 Video), indicating that NAM inhibited membrane resealing. The time course of dye uptake of the cells treated with NAM or control PBS after laser injury is shown in Fig 3c. After laser irradiation, vesicles under the injured membranes aggregated, fused with each other and formed a large convex of membranes patching the injured site in control cells (Fig 3d and 3e, S3 Video). However, in the presence of NAM, vesicles were aggregated under the injured membranes, but their fusion and/or attachment to the injured membranes were inhibited, resulting in the formation of concave membranes around the injured site (Fig 3d and 3e, S4 Video). Similar results were obtained when C2C12 cells were treated with 10 μM Ex527, a SIRT1 specific inhibitor (S5 and S6 Videos). Membrane protrusion after laser irradiation was observed in control cells after 4 min of laser exposure, whereas membrane repulsion was found in cells treated with NAM (Fig 3f).

*Sirt1-siRNA* was used to confirm the effects of SIRT1 on membrane repair (Fig 3g and 3h). Knockdown of SIRT1 by *Sirt1-siRNA* in C2C12 cells showed persisting intracellular entry of FM1-43 dye after laser injury (Fig 3i and 3j, S7 and S8 Videos). After laser irradiation, membrane repulsion was found in cells treated with *Sirt1-siRNA*, whereas membrane protrusion was detected in *Control-siRNA* treated cells (Fig 3k and 3l, S9 and S10 Videos). Treatment of cells with *Sirt1-siRNA* induced membrane repulsion, which was similar to those of cells treated with NAM (Fig 3m). Because SIRT1 regulates expressions of various genes, NAM and *Sirt1-siRNA* may affect gene expression levels of membrane repair proteins. NAM slightly affected mRNA levels of ANNA1 and ASM (Fig 3n), but *Sirt1-siRNA* had only a faint effect on the expression levels of ten membrane repair proteins and utrophin (Fig 3o). These results indicated that SIRT1 regulated the membrane repair process without transcriptional change of membrane-repair related genes. Whether SIRT1 is involved in membrane resealing in myotubes or not, we differentiated C2C12 cells to myotubes and examined their membrane injury and repair (Fig 3p). Caveolin 3 and nNOS, markers of myotubes, were expressed in the differentiated myotubes as shown in Fig 3q. SIRT1 expression levels of myotubes were comparable with those of C2C12 myoblast cells (Fig 3q). Similar to myoblast cells, membrane resealing after laser irradiation was constantly observed in differentiated myotubes (Fig 3r and 3s). When myotubes were treated with NAM, FM1-43 dye persistently entered intracellular species after laser irradiation (Fig 3r and 3s, S11 and S12 Videos).

## Discussion

In the present study, we showed that SIRT1-MKO mice had pathological and physiological characteristics similar to those of mild dystrophies, especially dysferlinopathy (Figs 1 and 2). High serum CK and LDH levels by exercise, indicated membrane fragility of muscles in SIRT1-MKO mice. EB uptake in the muscle after exercise in fact was significantly higher than that of WT mice (Fig 2e–2h). Although SIRT1-MKO mice had a high number of regenerating myofibers and lower number of middle-sized myofibers with high serum CK levels compared with those of WT mice, necrotic fibers, fibrosis and inflammatory changes were barely detected in their muscles (Fig 1e–1i). These phenotypes are common with clinical features of dysferlinopathy including limb girdle dystrophy type 2B and Miyoshi myopathy, which show minimal dystrophic change and decreased exercise performance with very high serum CK levels in their early stages [22, 23]. Interestingly, exercise in the early stage of dysferlinopathy accelerated the progression of disease [23]. Dysferlin is indispensable for membrane repair mechanism in which vesicles containing dysferlin aggregate and fuse beneath injured membranes [24]. We found that SIRT1 inhibition or knockdown disturbed membrane repair and inhibited vesicle aggregation and fusion at the injured site (Fig 3). To our knowledge, this is the first report to demonstrate the role of SIRT1 in membrane repair.

In the Western blot analysis, the intensity of SIRT1 band in the membrane fraction was less than that of cytosolic fraction (Fig 1b). This may be derived from that total amount of cytoplasmic SIRT1 is dominant in skeletal muscles and/or membrane SIRT1 is easily detached from plasma membranes under cellular fractionation experiments. nNOS, a member of dystroglycan complex, mainly localized beneath cellular membranes (Fig 1a). However, as shown in Fig 1b, nNOS was dominantly detected in the cytoplasmic fraction in the Western blot as reported by Chang et al. [25].

Ca^2+^ influx via damaged membranes stimulates aggregation of exocytic vesicles beneath injured membranes [1] and induces vesicles to form a membrane patch on the injured site by cross-linking them [26]. Treatment of cells with SIRT1 inhibitors or *Sirt1-siRNA* seemed to disturb intracellular vesicle fusion and/or attachment of vesicles to the injured membranes (Fig 3). A large patch formation on the wound site was also inhibited by NAM and *Sirt1-siRNA* (Fig 3d and 3k). Exocytosis is a mechanism to transport neurotransmitters and proteins out of the cells and participates in the provision of new membranes at the leading edge of migrating cells. Previously, we showed that SIRT1 is expressed in lamellipodium, a membrane protrusion of migrating cells, and is necessary for lamellipodium formation and migration of melanoma cells [27]. Contribution of SIRT1 on lamellipodium formation suggests that SIRT1 may be involved in cell membrane endocytosis and/or exocytosis [27]. Accordingly, SIRT1 has been demonstrated to positively regulate exocytosis-mediated hormone secretion [28]. In pituitary cells, SIRT1 positively regulates the exocytic release of thyroid-stimulating hormone via deacetylating and activating phosphatidylinositol-4-phosphate 5-kinase γ (PIP5Kγ) [29], which is involved in exocytosis of synaptic vesicles [30]. At present, it is not known whether PIP5Kγ is involved in membrane repair of C2C12 cells or not. Caveolin 1 and 3 are plasma membrane proteins which regulate endocytosis of dysferlin [31], and caveolin 3 interacts with MG53 and dysferlin to promote membrane repair [5]. Importantly, the scaffolding domain of caveolin 1, which is conserved between caveolin 1 and caveolin 3, directly binds SIRT1 [32]. Thus, caveolins may recruit SIRT1 beneath plasma membranes, where SIRT1 may deacetylate target protein(s) to reseal damaged membranes.

We have reported that a SIRT1 activator resveratrol decreases cardiac pathophysiology with extension of lifespan in δ-sarcoglycan-deficient TO-2 hamsters [18] and ameliorates muscular and cardiac pathophysiologies of dystrophin-deficient *mdx* mice [13–17]. SIRT1 overexpression in *mdx* mice ameliorates dystrophic phenotypes with decreased serum CK levels and muscle EB uptake [12]. At present, it is not known whether resveratrol or SIRT1 overexpression promotes membrane resealing or not.

SIRT1-MKO mice showed shorter hanging times and lower grasping power (Fig 2), indicating that SIRT1 plays some role in muscle strength as well. Reduction of muscle contraction activities has been reported in mice lacking MG53 [7] or ANNA1 [33], membrane repair proteins, although dysferlin-deficient mice showed normal contraction activities [34]. SIRT1 promotes mitochondrial biogenesis [8]. Accordingly, reduced mitochondrial function and decreased expression levels of mitochondrial proteins have been reported in muscles from whole-body SIRT1 knockout mice [11] and skeletal muscle-specific SIRT1 knockout mice [12], respectively. Thus, impaired mitochondrial function may affect muscle strength in SIRT1-MKO mice.

Our study indicates that SIRT1 plays role in membrane repair of skeletal muscle. Further studies of SIRT1 activation will reveal its effect on muscular dystrophies.

## Supporting information

S1 Fig HE staining of quadriceps from 12 months old SIRT1-MKO mice. (a) Immunofluorescence staining for SIRT1, dystrophin, caveolin 3 and nNOS in muscle sections from SIRT1-MKO mice at 6 months of age. (b) Arrowheads indicate fiber splitting. (c) Basophilic staining shows a regenerating fiber in a section of HE staining (arrow). (d) Immunofluorescence staining for SIRT1 (07–131, Merck Millipore) in muscle sections from WT and SIRT1-MKO mice at 6 months of age.
(TIFF)

S2 Fig Body weights of WT and SIRT1-MKO mice. Body weights of WT and SIRT1-MKO mice at 3 (a), 5 (b), 14 (c), and (d) 30 months old. n. s. not significant.
(TIFF)

S1 Video Movie of C2C12 cells treated with PBS. (AVI)

S2 Video Movie of C2C12 cells treated with 10 mM NAM. (AVI)

S3 Video Movie of C2C12 cells treated with PBS (High power field). (AVI)

S4 Video Movie of C2C12 cells treated with 10 mM NAM (High power field). (AVI)

S5 Video Movie of C2C12 cells treated with DMSO. (AVI)

S6 Video Movie of C2C12 cells treated with 10 μM Ex527. (AVI)

S7 Video Movie of C2C12 cells treated with *Control-siRNA*. (AVI)

S8 Video Movie of C2C12 cells treated with *Sirt1-siRNA*. (AVI)

S9 Video Movie of C2C12 cells treated with *Control-siRNA* (High power field). (AVI)

S10 Video Movie of C2C12 cells treated with *Sirt1-siRNA* (High power field). (AVI)

S11 Video Movie of C2C12 myotubes treated with PBS. (AVI)

S12 Video Movie of C2C12 myotubes treated with 10 mM NAM. (AVI)
